# Validating Disease Associations of Drug-Metabolizing Enzymes through Genome-Wide Association Study Data Analysis

**DOI:** 10.3390/genes15101326

**Published:** 2024-10-15

**Authors:** Evan Leskiw, Adeline Whaley, Peter Hopwood, Tailyn Houston, Nehal Murib, Donna Al-Falih, Ryoichi Fujiwara

**Affiliations:** 1 Department of Pharmaceutical Sciences, College of Pharmacy, Northeast Ohio Medical University, Rootstown, OH 44272, USA; 2 College of Medicine, Northeast Ohio Medical University, Rootstown, OH 44272, USA; 3 College of Arts and Sciences, Cleveland State University, Cleveland, OH 44115, USA

**Keywords:** pharmacogenomics, drug metabolism, enzymes, UDP-glucuronosyltransferase, genome-wide association study

## Abstract

Background and Objectives: Phase I and phase II drug-metabolizing enzymes are crucial for the metabolism and elimination of various endogenous and exogenous compounds, such as small-molecule hormones, drugs, and xenobiotic carcinogens. While in vitro and animal studies have suggested a link between genetic mutations in these enzymes and an increased risk of cancer, human in vivo studies have provided limited supportive evidence. Methods: Genome-wide association studies (GWASs) are a powerful tool for identifying genes associated with specific diseases by comparing two large groups of individuals. In the present study, we analyzed a GWAS database to identify key diseases genetically associated with drug-metabolizing enzymes, focusing on UDP-glucuronosyltransferases (UGTs). Results: Our analysis confirmed a strong association between the *UGT1* gene and hyperbilirubinemia. Additionally, over ten studies reported a link between the *UGT1* gene and increased low-density lipoprotein (LDL) cholesterol levels. *UGT2B7* was found to be associated with testosterone levels, total cholesterol levels, and vitamin D levels. Conclusions: Despite the in vitro capability of UGT1 and UGT2 family enzymes to metabolize small-molecule carcinogens, the GWAS data did not indicate their genetic association with cancer, except for one study that linked *UGT2B4* to ovarian cancer. Further investigations are necessary to fill the gap between in vitro, animal, and human in vivo data.

## 1. Introduction

Drug-metabolizing enzymes are a group of enzymes that can metabolize small-molecule drugs. Enzymes that catalyze the oxidation, reduction, and hydrolysis of drugs are called phase I drug-metabolizing enzymes. Meanwhile, phase II drug-metabolizing enzymes catalyze conjugation reactions, such as glucuronidation and sulfation. Cytochrome P450 (CYP) family enzymes are one of the major types of phase I drug-metabolizing enzymes [1]. CYP3A4 is highly expressed in the liver, which is the most important tissue for drug metabolism, and contributes to nearly 50% of phase I drug metabolism [2]. Another phase I enzyme is carboxylesterase (CES), which is responsible for the hydrolysis of many drugs. UDP-glucuronosyltransferase (UGT) and sulfotransferase (SULT) catalyze the glucuronidation and sulfation of drugs, respectively. Phase I and II metabolites are typically more hydrophilic and pharmacologically less active compared to the parent form of the drug; therefore, phase I and II drug-metabolizing enzymes are crucial for the metabolism and elimination of small-molecule drugs.

In addition to drugs, drug-metabolizing enzymes are also capable of metabolizing various endogenous and exogenous small molecules, including bilirubin, thyroxine, estradiol, vinyl chloride, heterocyclic amines, and other xenobiotic carcinogens [3]. Because of the diverse function of these enzymes, it has been hypothesized that genetic mutations and deficiencies in the drug-metabolizing enzyme genes are associated with various diseases, such as cancer, radiation-induced secondary malignancies, colitis, jaundice, alcoholism, and depressive disorder (Table 1) [4,5,6,7,8,9,10,11,12,13,14,15]. For some cases, the molecular mechanism that explains the association of enzyme function with the onset of disease has been fully supported by a series of in vitro, animal, and human in vivo data. However, human in vivo studies have provided limited supportive evidence for most of the hypothesized diseases.

Genome-wide association studies (GWASs) are a powerful tool for identifying genes associated with specific diseases by comparing two large groups of individuals. Typically, the data are presented as a Manhattan plot, which is a scatter plot that shows the significance of the gene variations by chromosome location. For instance, a study that compares the genetic differences between two populations, one with breast cancer and the other without, can identify a genetic mutation that is specific to the breast cancer group. There are several free, publicly available databases that summarize GWAS data. Each database has their own features. In GWAS Catalog (https://www.ebi.ac.uk/gwas, accessed on 10 October 2024), by typing a gene name, diseases or traits that are associated with the gene will be shown. Meanwhile, in GWAS Atlas (https://atlas.ctglab.nl), genes that are associated with diseases or traits will be shown by choosing a disease or a trait. Either way, the data are presented without a hypothesis, as it only displays a specific health trait, the genetic region associated with that trait, and the statistical significance associated with the correlation.

In the present qualitative study, we analyzed a GWAS database to identify key diseases genetically associated with drug-metabolizing enzymes, focusing on UGT enzymes.

## 2. Methods

Acquisition of all data was achieved by reclassifying compiled studies from the GWAS Catalog (https://www.ebi.ac.uk/gwas), which is a database that collects human in vivo GWAS data. Published GWAS data concerning the *UGT* (*UGT1* and *UGT2*), *CYP* (*CYP2C19*, *CYP1A2*, *CYP2E1*, *CYP2B6*, *CYP2C8*, *CYP2A6*, *CYP2C9*, *CYP2J2*, and *CYP3A5*), *CES* (*CES1* and *CES2*), and *SULT* (*SULT1*, *SULT2*, *SULT4*, and *SULT6*) genes were compiled into spreadsheets. The diseases/traits that were associated with the genes and the number of the reports were summarized into a table. A strong association was defined as 10 or more GWAS reports linking a specific gene to a disease or trait, while a moderate association was defined as 3 or more GWAS reports indicating a link. Data collection and analysis were conducted between January and August 2024. The spreadsheets are not publicly available; however, the authors are open to sharing the data with individuals who provide a reasonable request.

## 3. Results

### 3.1. UGT1

The *UGT1* gene encodes various UGT1A family isoforms from UGT1A1 to UGT1A10 by the exon-sharing mechanism. GWAS Catalog contained over 100 studies that reported a genetic association between the *UGT1* gene and various diseases. A strong association was observed between the *UGT1* gene and jaundice (hyperbilirubinemia). Additionally, over ten studies reported a link between the *UGT1* gene and increased low-density lipoprotein (LDL) cholesterol levels. The serum levels of certain endogenous and exogenous metabolites were also shown to be linked to the *UGT1* gene. Miscellaneous diseases and traits that were associated with *UGT1* were total cholesterol levels, high-density lipoprotein (HDL) levels, body fat percentage, cholelithiasis, bone mineral density, biliverdin levels, circulating cell-free DNA levels, and response to a drug (dolutegravir) (Table 2).

### 3.2. UGT2

UGT2 family isoforms are encoded by the individual genes. In this study, we analyzed the *UGT2B4*, *UGT2B7*, *UGT2B10*, *UGT2B11*, *UGT2B15*, *UGT2B17*, and *UGT2B28* genes.

There were only 15 studies in the GWAS Catalog that reported diseases and traits that were linked to *UGT2B4*. One of the most common traits that was associated with this gene was the concentration of small molecules including metabolites such as androsterone glucuronide, *X*-12844, *X*-24418, and *X*-24947. *UGT2B4* was also associated with the serum vitamin D level. Miscellaneous diseases and traits that were associated with *UGT2B4* were bacteremia, color vision defects, and malignant ovarian tumors (Table 2).

There were 48 studies in the GWAS Catalog that reported diseases and traits that were linked to *UGT2B7*. The blood testosterone level was the most common trait that was associated with the *UGT2B7* gene. Eight studies reported a genetic association between *UGT2B7* and serum vitamin D levels. Total cholesterol levels seem to be moderately associated with *UGT2B7*. *UGT2B7* was also associated with blood levels of drugs and metabolites such as AR-C124910XX, dihydroxydocosapentaenoic acid, eicosanoid 13S-HpOTrE, estradiol, and glycochenodeoxycholate glucuronide. Miscellaneous diseases and traits that were associated with *UGT2B7* include body height, diastolic blood pressure, systolic blood pressure, LDL levels, medication use, and *γ*-glutamyl transferase levels (Table 2).

There were only 80 studies in the GWAS Catalog that reported diseases and traits that were linked to *UGT2B10*. One of the most common traits that was associated with this gene was blood measurements including cotinine, bilirubin, blood plasma, N-desmethylclozapine, and red blood cell width. *UGT2B10* was also associated with the lipid/metabolic measure. Mean corpuscular hemoglobin concentration/volume, sex hormone-binding globulin, and testosterone were moderately associated with this gene. Miscellaneous diseases and traits that were associated with *UGT2B10* were nose morphology, apolipoprotein A1 measurements, serum *γ*-glutamyl transferase measurements, body height, C-X-C motif chemokine 10 measurements, cortolone glucuronide measurements, and deoxycholic acid (Table 2).

There were only 13 studies in the GWAS Catalog that reported diseases and traits that were linked to *UGT2B11*. Several blood plasma and metabolite measurements were associated with *UGT2B11*. Four studies reported an association between *UGT2B11* and vitamin D or cholesterol measures. *UGT2B11* was also associated with disease or disease progression. Miscellaneous diseases and traits that were associated with *UGT2B11* were testosterone, neuritic plaques, and alkaline phosphatase measures (Table 2).

We further analyzed *UGT2B15*, *UGT2B17,* and *UGT2B28* to determine whether they were genetically linked to cancer. There were 88, 188, and 7 studies in the GWAS Catalog that reported diseases and traits linked to *UGT2B15*, *UGT2B17*, and *UGT2B28,* respectively. None of the data showed their potential genetic linkage to cancer (Table 2).

### 3.3. Other Phase I and II Enzymes

CYP is a superfamily enzyme. Among a number of functional CYP family enzymes, CYP3A4 contributes the most to the metabolism of clinically used drugs. In addition to xenobiotic molecules, various endogenous compounds are substrates of CYP3A4. As of June 2024, the GWAS Catalog contains 146 entries that report a genetic association between *CYP3A4* and a disease/trait. Most of the reports linked *CYP3A4* with blood or urine levels of xenobiotics, hormones, and their metabolites. Our qualitative study showed that *CYP3A4* was closely associated with the levels of testosterone, estradiol, androsterone, and their metabolites. CYP is a phase I enzyme; therefore, it does not catalyze a series of conjugation reactions. However, various sulfate and glucuronide metabolites were identified as traits that were linked to *CYP3A4*. Such unique traits include the levels of androsterone sulfate, 16a-hydroxy dehydroepiandrosterone 3-sulfate, dehydroepiandrosterone sulfate, and etiocholanolone glucuronide. Miscellaneous diseases and traits that were associated with *CYP3A4* include cytokine levels, post-traumatic stress disorder (PTSD), heel bone mineral density, height, offspring birth weight, lymphocyte count, C-reactive protein levels, and triglyceride levels.

In addition, CYP3A4, CYP1A2, CYP2A6, CYP2B6, CYP2C8, CYP2C9, CYP2C19, CYP2D6, CYP2E1, CYP2J2, and CYP3A5 play a key role in drug metabolism in the liver. Like CYP3A4, these CYP family enzymes are also capable of metabolizing various endogenous and exogenous compounds. The GWAS Catalog contained more than 120 entries that associated *CYP1A2* with a disease/trait. Coffee (caffeine) consumption, blood pressure, medication use, estimated glomerular filtration rate (eGFR), urinalysis test results, body composition, drug and metabolite concentrations, and beverage and food consumption were the common traits that were associated with *CYP1A2*. Three studies reported a genetic association between *CYP1A2* and lung cancer. Minor traits that were linked to *CYP1A2* included serum albumin levels, red blood cell count, platelet distribution width, coronary heart disease, cardiovascular disease, brain morphology, and peak expiratory flow.

In the GWAS Catalog, more than 230 entries reported an association between *CYP2A6* and a disease/trait. The most common trait that was associated with *CYP2A6* was related to smoking behaviors, such as tobacco use, cigarettes smoked per day, nicotine metabolite ratio, smoking status, cotinine levels, nicotine dependence, and smoking cessation. Liver enzyme levels and lipid test values were also highly associated with *CYP2A6*. Eighteen studies reported a link between *CYP2A6* and lung cancer. Lung function test values were also moderately associated with *CYP2A6*. In addition to various serum and urine levels of metabolites, miscellaneous traits that were linked to *CYP2A6* included chronotype, vaginal microbiome relative abundance, parental longevity, carotid intima media thickness, and BMI.

The GWAS Catalog reported 20 traits that were associated with *CYP2B6*. The major trait that was linked to *CYP2B6* was the levels of xenobiotics including caffeine and pesticides. Smoking behaviors and mastocytosis were moderately associated with the gene. Minor traits that were associated with *CYP2B6* were HDL cholesterol levels and neuritic plaques.

There were 61 entries that reported a genetic association between *CYP2C8* and a disease/trait in the GWAS Catalog. The most common type of traits that were linked to *CYP2C8* were levels of endogenous and exogenous compounds and their metabolites, including testosterone, androstenediol, and warfarin. Minor traits that were linked to this gene were total cholesterol levels, LDL levels, osteonecrosis of the jaw, gout, immune response to smallpox vaccine, appendicular lean mass, tuberculosis, and response to amphetamines.

In the GWAS Catalog, 71 entries reported a genetic association between *CYP2C9* and a disease/trait. The levels of various xenobiotics and their metabolites were associated with *CYP2C9*. One of the unique traits that was observed in multiple studies was the levels of fatty acids and their metabolites, such as omega-3 and omega-6 fatty acids. Blood pressure was also moderately associated with *CYP2C9*. Miscellaneous traits that were linked to this gene were appendicular lean mass, glucocorticoid receptor gene expression, waist circumference, lymphocyte count, and liver enzyme levels.

There were 32 entries in the GWAS Catalog that showed a genetic link between *CYP2C19* and a disease/trait. The levels of drugs and drug metabolites were the most common traits that were associated with *CYP2C19*. Multiple studies showed a genetic association between *CYP2C19* and blood pressure. Cholesterol levels were also moderately linked to *CYP2C19*. Minor traits that were associated with this gene included migraine, appendicular lean mass, and lymphocyte count.

There were only a handful of genome-wide studies that reported traits associated with *CYP2D6*, *CYP2E1*, and *CYP2J2*. Traits that were linked to *CYP2D6* were the levels of metabolites, response to a drug, insomnia, and brain function. Gout, obesity, and acute myeloid leukemia were some of the traits that were associated with *CYP2E1*. *CYP2J2* was associated with attention deficit hyperactivity disorder (ADHD), aging, educational attainment, and levels of vitamin D, dopamine metabolites, and phenol sulfate.

The GWAS Catalog reported 25 traits that were associated with *CYP3A5*. Many studies showed a genetic association between *CYP3A5* and levels of drugs and metabolites such as tacrolimus, metabolomic lactone sulfate, eicosanoids, and methionine sulfone. Several studies reported platelet count, neutrophil count, and lymphocyte count as the traits that were associated with *CYP3A5*.

CES1 and CES2 are responsible for the hydrolysis of many drugs. In addition to *CES1* and *CES2*, we analyzed the *CES3* and *CES4* genes to see whether genetic mutations of *CES* were associated with diseases or traits. There were 12 entries in the GWAS Catalog that showed a genetic link between *CES1* and a disease/trait. The most common trait that was associated with *CES1* was cholesterol levels. Various studies showed a genetic link to an increase or decrease in the levels of total cholesterol, LDL, and HDL, depending on the type and location of the mutation. Minor traits that were associated with this gene included metabolite concentration, protein measurement, response to a drug, and QRS duration. In the GWAS Catalog, 29 entries reported a genetic association between *CES2* and a disease/trait. Interestingly, all the traits that were associated with the *CES2* gene were related to a lipid panel. Such traits included total lipid levels, free cholesterol levels, cholesteryl ester levels, HDL cholesterol levels, triglyceride-to-total lipid ratio, phospholipid-to-total lipid ratio, and apolipoprotein A1 levels. Thirteen genome-wide studies reported a genetic association between *CES3* and a trait. The most common trait that was associated with *CES3* was blood counts, such as red blood cell count, white blood cell count, and monocyte count. A trait that was moderately associated with this gene was heel bone mineral density. All the reported traits associated with the *CES4A* gene were related to a blood count. Depending on the type and location of the mutation, *CES4A* was associated with either an increase or decrease in the levels of hemoglobin, red blood cells, monocytes, and white blood cells.

In this study, we focused on *SULT1A1*, *SULT1A2*, and *SULT1E1* and analyzed their genetic associations with diseases and traits. They are some of the key SULT family enzymes that are functional in the liver, metabolizing various endogenous and exogenous substrates including carcinogens. There were 46 GWAS studies reporting a genetic association between *SULT1A1* and a disease/trait. One of the common traits that was associated with *SULT1A1* was related to brain function, which was reported as intelligence, general cognitive ability, educational attainment, and brain volumes. The GWAS Catalog also showed genetic associations between *SULT1A1* and the levels of certain metabolites, such as ascorbic acid 3-sulfate and hydroquinone sulfate. Multiple studies reported an association between *SULT1A1* and obesity-related traits including body fat percentage and body mass index (BMI). Miscellaneous diseases and traits that were associated with *SULT1A1* were risk-taking tendency, allergic rhinitis, alcohol frequency, red blood cell count, tonsillectomy, and mosquito bite size. Only five studies reported a genetic linkage between *SULT1A2* and a disease/trait. Two studies identified externalizing behavior and risk-taking behavior as traits that were linked to *SULT1A2*. Three studies demonstrated a genetic association between *SULT1A2* and levels of various compounds such as X-12707, ascorbic acid 3-sulfate, and 3-methoxycatechol sulfate. There were also only five studies reporting a genetic linkage between *SULT1E1* and a disease/trait. Three studies identified *SULT1E1* as one of the genes linked to heel bone mineral density. Two other studies linked *SULT1E1* to sex hormone-binding globulin levels.

### 3.4. Types of Variants

Overall, most of the variants were mutations in introns, and significant variants like missense mutations were minor. In fact, approximately 75%, 20%, and 5% of the reported genetic variations of *UGT1* that were linked to diseases/traits (Table 2) were mutations in introns, 3′ untranslated region variants, and missense mutations, respectively.

## 4. Discussion

UGT1A1 is the sole enzyme capable of metabolizing bilirubin, which is the end product of heme catabolism. A genetic deficiency or inhibition of UGT1A1 results in elevated serum bilirubin levels, leading to hyperbilirubinemia, commonly known as jaundice. Various in vitro and in vivo studies in both animals and humans have associated UGT1A1 function with serum bilirubin levels [7,16]. In the present study, our analysis confirmed the strong association between the *UGT1A1* gene and hyperbilirubinemia, highlighting the effectiveness of GWASs in validating gene–disease associations.

Interestingly, our analysis of GWAS data revealed multiple links between UGT genes and various diseases/traits, including increased low-density lipoprotein (LDL) cholesterol levels, vitamin D levels, color vision defects, and bone mineral density (Table 2). However, it is important to note that these associations do not necessarily imply that UGTs are directly and significantly involved in these diseases and traits. While they might be, caution is required when interpreting the data. Firstly, other genes may also contribute to the specific disease/trait. For example, the study that identified *UGT2B4* as one of the genes associated with color vision defects also identified 882 other genes linked to the disease [17]. Therefore, UGT2B4′s contribution to the disease might be minimal. Secondly, the mechanism linking UGT2B4 to color vision defects remains unknown. In this case, the reported genetic variant of *UGT2B4* is located in an intron (rs188529050), which does not directly alter the amino acid sequence or protein structure. However, it is possible that the intronic mutation affects transcription and splicing, leading to significant changes in enzyme activity. Other than the GWAS data, there was no available in vitro or animal data that linked UGT2B4 to color vision defects either. While GWAS data are generally accurate and confirm that the *UGT2B4* gene is genetically linked to color vision defects, it is unlikely that the induction or inhibition of UGT2B4 can prevent or accelerate disease onset. Further research is needed to elucidate the underlying mechanisms and to confirm the extent of UGT2B4′s involvement in these traits.

Another interesting observation was that our study did not find many genetic associations between UGTs and cancer despite numerous publications suggesting that mutations in UGT genes can increase cancer risk (Table 1). This discrepancy can be explained by several possibilities. First, the expression of drug-metabolizing enzymes, including UGTs, is highly regulated by various transcription factors such as the pregnane X receptor (PXR), the constitutive androstane receptor (CAR), the aryl hydrocarbon receptor (AhR), and nuclear factor erythroid 2-related factor 2 (NRF2) [18,19,20]. Activators of these transcription factors can be natural compounds found in food and supplements. Additionally, a group of small molecules can inhibit enzyme reactions. Factors such as gender, diet, age, drinking, and smoking also affect the function of drug-metabolizing enzymes [21,22,23,24,25]. These external and environmental factors, collectively known as nurture, might contribute to the observed discrepancy. Second, other metabolic or excretion pathways might contribute more significantly to the overall clearance of carcinogens. While UGTs are undoubtedly capable of metabolizing small-molecule carcinogens, this does not necessarily mean that glucuronidation by UGTs is the primary metabolic and detoxification pathway for all carcinogens. Third, the sample size in our study might still be too small. It is also reasonable to question whether the small-molecule carcinogens listed in Table 1 are the primary causes of cancer. UGTs might be identified as genes associated with cancer development in a GWAS that specifically analyzes the effect of long-term exposure to UGT substrate carcinogens on cancer development.

In vitro and animal studies have also linked mutations in the *CYP*, *CES*, and *SULT* genes to cancer because these enzymes can metabolize small-molecule carcinogens [26,27,28]. Multiple GWAS analyses showed genetic associations between CYP1A2 and CYP2A6 and cancer. CYP1A2 is a key enzyme involved in the bioactivation of tobacco-specific nitrosamines, while CYP2A6 is the main enzyme that metabolizes nicotine and tobacco-related carcinogens. Individuals who are poor metabolizers of nicotine tend to smoke less, while those who metabolize it more efficiently tend to smoke more. This connection likely explains the GWAS findings linking these enzymes to lung cancer. However, it is possible that smoking behavior is the primary cause of lung cancer, with genetic mutations in *CYP1A2* and *CYP2A6* being secondary factors in its development. Whether *CYP1A2* and *CYP2A6* are genetically linked to lung cancer in nonsmokers remains unclear. Aside from CYP1A2 and CYP2A6, no significant cancer associations were found for the other phase I and phase II enzymes examined in this study. Instead, GWAS data showed associations between some enzymes and other diseases, including PTSD, ADHD, high cholesterol, high blood pressure, and obesity. As discussed earlier, the molecular mechanisms behind these associations with drug-metabolizing enzymes are still not fully understood. Therefore, careful consideration is necessary when translating GWAS data related to these conditions.

In summary, GWASs have significantly contributed to the identification of genetic causes of diseases, leading to the development of accurate gene diagnostic systems that predict diseases, including cancer. To our knowledge, this is the first qualitative study that identified diseases and traits that were associated with drug-metabolizing enzyme genes using an analysis of a GWAS database. Previous research publications suggested a potential increased probability of developing cancer in individuals with mutations in *UGT* genes. However, our study did not find genetic associations between UGTs and cancer, except for one study that linked *UGT2B4* to ovarian cancer. While GWAS data can provide valuable insights, further investigations with larger sample sizes and a focus on long-term exposure to specific carcinogens are necessary to fully understand the role of UGTs in cancer development.

## Figures and Tables

**Table 1:** Examples of hypothesized diseases and traits linked to drug-metabolizing enzyme genes.

Enzymes	Disease/Trait	Mechanism
Drug-metabolizing enzymes in general	Radiation-induced secondary malignancies	Common genetic polymorphisms in drug-metabolizing enzymes that result in impaired detoxification of chemotherapy or inefficient repair of drug- or radiation-induced genetic damage may lead to increased risk of a second cancer [4].
UGT	Bladder cancer	Carcinogens including benzidine are metabolized by UGTs [5].
UGT	Colitis	UGTs prevent accumulation of toxic bile acids in the colon [6].
UGT1A1	Hyperbilirubinemia	Bilirubin is solely metabolized by UGT1A1 [7].
UGT	Lung cancer	Lung cancer-causing polycyclic aromatic hydrocarbons (PAHs) are metabolized by UGTs [8].
UGT2	Cancer	UGT2 metabolizes tobacco-specific nitrosamines [9].
UGT1	Cancer	2-Amino-1-methyl-6-phenylimidazo [4,5-b]pyridine (PhIP) is metabolized by UGT1s [10].
UGT	Thyroid tumors	UGTs are involved with T3 and T4 metabolism [11].
UGT	Breast cancer	Bisphenol-A is metabolized by UGT [12].
UGT	Renal cancer	N-Hydroxyphenacetin O-glucuronide is a promutagen [13].
UGT	Breast cancer	UGTs are involved with estrogen metabolism [14].
UGT1A6	Depressive disorder	UGT1A6 metabolizes serotonin [15].
UGT	Smoking	UGT metabolizes nicotine [15].
UGT	Alcoholism	UGT metabolizes ethanol [15].

**Table 2:** Reported diseases and traits genetically linked to *UGT1* and *UGT2* genes.

Gene	Location	Associated Diseases/Traits	No. of Reports
*UGT1*	2q37	Bilirubin levels	45
		Blood drug/metabolite levels	25
		Cholesterol traits (LDL increase, body fat %, total)	11
		Miscellaneous (bone mineral density, biliverdin, level of circulating cell-free DNA)	29
			Total 110
*UGT2B4*	4q13	Blood metabolite levels	9
		Vitamin D levels	3
		Miscellaneous (bacteremia, color vision defect, malignant ovarian tumor)	3
			Total 15
*UGT2B7*	4q13	Blood testosterone levels	10
		Blood vitamin D	8
		Cholesterol-related (increase/decrease and total levels)	8
		Blood drug/metabolite levels	7
		Miscellaneous (body height, diastolic and systolic blood pressure, neurofibrillary tangles, medications, *γ*-glutamyl transferase measure)	15
			Total 48
*UGT2B10*	4q13	Blood measurements (cotinine, bilirubin, metabolites, N-desmethylclozapine, red blood cell width)	29
		Lipid/Metabolic measure (total cholesterol, HDL, LDL, body fat, etc.)	18
		Mean corpuscular hemoglobin concentration/volume	10
		Sex hormone-binding globulin	9
		Testosterone	5
		Miscellaneous (nose morphology, apolipoprotein A1 measurement, serum *γ*-glutamyl transferase measurement, body height, C-X-C motif chemokine 10 measurement, cortolone glucuronid measurement, deoxycholic acid)	9
			Total 80
*UGT2B11*	4q13	Blood measurements (vitamin D, cholesterol, testosterone, etc.)	9
		Disease/Disease progression measure	2
		Miscellaneous (neuritic plaque, alkaline phosphatase measure)	2
			Total 13
*UGT2B15*	4q13	Blood metabolite measurements	61
		Hormone-related measurements	53
		Triglyceride/Lipoprotein content (high/low density, phospolipids, cholesterol, omega-3 fatty acids, non-high density HDL, body fat)	43
		Miscellaneous (serum *γ*-glutamyl transferase, cotinine, alkaline phosphatase, deoxycholic acid, apoliprotein A1, cortolone glucuronide, HMG CoA reductase)	31
			Total 188
*UGT2B17*	4q13	Hormone levels (steroid/steroid hormones, sex hormone-binding globulin)	35
		Cholesterol-related traits (free phospholipids, cholesterol, HDL/LDL, omega-3 fatty acids, triglycerides, body fat)	33
		Blood metabolites	9
		Alkaline phosphatase	3
		Miscellaneous (IGF-1, DNA repair protein, cholic acid glucuronide, deoxycholic acid, 1-stearoyl-2-docosahexaenoyl-*sn*-glycero-3-phosphocholine, free androgen index)	8
			Total 88
*UGT2B28*	4q13	Cell membrane components (Phosphatidylcholine/Sphingomyelin measurement)	3
		Total cholesterol levels	2
		Miscellaneous (lean body mass, vitamin D measure)	2
			Total 7

## Data Availability

The original GWAS data can be found at https://www.ebi.ac.uk/gwas (accessed on 10 October 2024).
